# Deconstructing the notion of “global health research partnerships” across Northern and African contexts

**DOI:** 10.1186/s12910-018-0280-7

**Published:** 2018-06-15

**Authors:** Lara Gautier, Isidore Sieleunou, Albino Kalolo

**Affiliations:** 1 School of Public Health, University of Montreal, Montreal, Canada; 2 University of Montreal Public Health Research Institute, Montreal, Canada; 3 Centre d’études en sciences sociales sur les mondes africains, américains et asiatiques (UMR 245), Institut de Recherche pour le Développement, Université Sorbonne Paris Cité, Paris, France; 4 International Development Research Centre, Yaoundé, Cameroon; 5 St. Francis University College of Health and Allied Sciences, Ifakara, Tanzania

**Keywords:** Global health, Partnership, Equity, Research ethics, Sub-Saharan Africa

## Abstract

**Background:**

Global health conceives the notion of partnership between North and South as central to the foundations of this academic field. Indeed, global health aspires to an equal positioning of Northern and Southern actors. While the notion of partnership may be used to position the field of global health morally, this politicization may mask persisting inequalities in global health. In this paper, we reflect on global health partnerships by revisiting the origins of global health and deconstructing the notion of partnership. We also review promising initiatives that may help to rebalance the relationship.

**Results and Discussion:**

Historical accounts are helpful in unpacking the genesis of collaborative research between Northerners and Southerners – particularly those coming from the African continent. In the 1980s, the creation of a scientific hub of working relationships based on material differences created a context that was bound to create tensions between the alleged “partners”. Today, partnerships provide assistance to underfunded African research institutions, but this assistance is often tied with hypotheses about program priorities that Northern funders require from their Southern collaborators. African researchers are often unable to lead or contribute substantially to publications for lack of scientific writing skills, for instance. Conversely, academics from African countries report frustrations at not being consulted when the main conceptual issues of a research project are discussed. However, in the name of political correctness, these frustrations are not spoken aloud. Fortunately, initiatives that shift paternalistic programs to formally incorporate a mutually beneficial design at their inception with equal input from all stakeholders are becoming increasingly prominent, especially initiatives involving young researchers.

**Conclusion:**

Several concrete steps can be undertaken to rethink partnerships. This goes hand in hand with reconceptualizing global health as an academic discipline, mainly through being explicit about past and present inequalities between Northern and Southern universities that this discipline has thus far eluded. Authentic and transformative partnerships are vital to overcome the one-sided nature of many partnerships that can provide a breeding-ground for inequality.

## Background

Global health conceives of the notion of partnership between North and South as central to the foundations of this academic field. Indeed, embracing the popular social justice discourse that is inherent to ethics, global health aspires to an equal positioning of Northern and Southern actors. In other words, partnership in its current form supposedly rests on the autonomy and independence of each partnering entity [1]. Northern actors encompass what is also generally referred to as “Western actors”, i.e., institutions and individuals representing the USA and Canada, Europe (European Union and European Free Trade Association member states), Australia, and New Zealand, whereas Southern actors represent low- and middle-income countries. For the purpose of this article, we focus our analysis on sub-Saharan African countries.

Some authors have posited that “[p]erhaps in response to… postcolonial anxieties, the term ‘partnership’ has emerged as a key word within this new arena or ‘social world’ of global health” [2]. However, the meaning of global health partnerships is far from straightforward. Today, a partnership may refer to a small-scale community-based intervention in a sub-Saharan African country as much as to a transnational structure administering research projects between Northern and African universities. Perhaps for being such a vague concept, global health partnerships have so far proved unable to redress the widespread ongoing inequalities [2, 3]. Scientific partnerships in particular, which mobilize researchers from different disciplines, geographical spaces, and cultures, still involve numerous and complex ethical issues [4]. Gross disparities in access to good quality training, funding opportunities (fellowships, travel grants to attend international conferences, etc.), and publishing opportunities represent potential threats to social justice between researchers [5]. A review of peer-reviewed literature on health financing in Burkina Faso found that home institutions of first authors were North-based in 95% of the papers [6]. Another search published in 2008 using the PubMed database found that 87% of articles by authors affiliated with university global health programs were from North America [7].

Crane argues that global health leaders employ the notion of partnership to position the field of global health morally by allying it with an ethics of equity [2]. From this perspective, partnerships would appear as opportune instruments for showing good faith and equitable conduct, i.e., the showcase for equity and social justice that were lacking in the past. Deconstructing the expression “global health partnership” is therefore essential for anyone who conceives of it as something genuinely oriented towards more balance between North and South. One cannot, however, deconstruct the concept of partnership in global health without first touching upon the origins of the field of global health itself, as we attempt in the first of three sections below. In the second section, we review of the notion of partnership as it is applied in global health by providing concrete examples of how and why global health research and practice can translate into blatantly unequal partnerships. However useful their perspectives are, authors such as Crane do not go so far as to suggest what a conception of global health not marked by colonialism might look like, and by extension, what “really equal” partnerships would imply. In the last section of this article, we offer a few general and concrete ideas on how to fill these gaps.

## Results and Discussion

### Revisiting the origins of global health

Deconstructing implies “examin[ing] in order to reveal the basis or composition of often with the intention of exposing biases, flaws, or inconsistencies” [8]. Originally, global health was built around the need to shift from an explicitly unequal give–take relationship towards a more mutually beneficial cooperation between Northern institutions and their Southern counterparts. Indeed, some argue that the moral frameworks underlying tropical medicine and international health (the antecedents of global health), inherited from colonialism, may have justified inequality by “establishing a hierarchy between Europe and Africa, and between science and its subjects, through idioms of, respectively, scientific racism, paternalism, or more recently, solidarity and aid” [1]. Recent times have seen a movement away from such paternalistic and top-down modes of operation, which characterized international health and tropical medicine [9], towards a new academic field that would effectively address inequalities, that is, global health.

Global health is defined as “an area for study, research, and practice that places a priority on improving health and achieving equity in health for all people worldwide” [9]. However, this definition of global health is itself controversial. Indeed, it officially represents a consensus reached during the 2008 inaugural meeting of the Consortium of Universities for Global Health (CUGH)—representing mainly Johns Hopkins University and the University of Washington. Yet during that meeting (at which only five participants represented Southern countries), a tension arose among participants over what global health actually means. Participants therefore acknowledged that “global health is a Northern concept” and that “for the academic institutions of the Global South, everyday public health, medical and nursing education and practices constitute ‘global health’” ([10], cited in 2). The postcolonial power dynamics of the term and its precursors (tropical medicine and international health) were not discussed; if they had been, participants would probably have come out with a different framing and conceptualization. Issues transcending traditional borders, such as medical tourism and waves of migrants and refugees, were also absent from the discussions. The conversation also avoided issues pertaining to biopolitical public health, which extends beyond any one nation and is deeply constitutive of modern nation-building ([10], cited in 2). Yet exploring how practices of global health contribute to states’ exercise of power is becoming critical. Traditionally the realm of individual states, public health is increasingly a concern of the global community. For instance, infectious diseases have moved from the realm of health issue to that of international security threat. This new framing may raise concerns over the nature of biopolitical citizenship [11]. Avoiding these issues made it possible to reach a seemingly perfect consensus in the published definition of global health. Despite attempts to re-open the debate about what constitutes global health [12, 13], since 2009 no consensus has been reached on the matter. As a result, Kopan’s definition [9] remains the most widely cited in the literature.

### Thirty years of academic partnership inequality?

Partnerships have been defined as “contextually relevant peer-to-peer collaborations which offer a platform for sharing knowledge and growing expertise globally, working towards a common goal, across disciplines and perspectives” [14]. The principles of partnership as described here appear very similar to those of global health [9]. In their seminal article, Koplan et al., however, simply referred to a “shift in philosophy… that emphasizes the mutuality of real partnership” [9]. The substance of this “real partnership” remains to be defined. Despite promises of more equality, the relationship has remained roughly the same, with, at the macro level, North to South transfers of financial or material resources and “capacity-building” initiatives in the disadvantaged South [15]. In the following paragraphs, we use practical examples from meso and micro levels to illustrate our macro-level arguments. We explore how institutions (macro), research teams (meso), and individuals (micro) tend to reproduce the imbalance of power.

#### Institutional and individual inequalities: unpacking the “unknown knowns” in partnerships

From a macro perspective, historical accounts are helpful in unpacking the genesis of collaborative research between Northerners and Southerners, particularly from the African continent. Anthropological evidence explains that such collaborations were “shaped by declining standards and scientific possibilities in ordinary, entirely state-funded university departments, hospitals, and laboratories across Africa…, caused by economic and political crisis and privatizations since the late 1970s” [1]. Consequently, technical, organizational, and financial support from “outside partners” were extensively needed. This created a scientific hub of working relationships based on material differences, a context that could not but lead to tensions between partners and alleged abuse of power [16, 17]. And how could this pattern change, when government’s disinterest in investing in universities and social science programs is blatant, as it is in sub-Saharan countries [18]? Yet, in spite of obvious material discrepancies, the language of “collaborative partnership”, often “compounded by the fictions of official government policy and those of the global development partners” [1], insists that North-based research institutions engage Southern partners as equals.

At the meso level, for instance, it is not politically correct to write that one party is less able than the other to achieve clinical aims, since it would break the collaborative consensus. This alleged consensus rests on a clear division of tasks that is formally accepted by research teams from North and South: i) researchers from the North (the P.I.s) write proposals, often with little involvement from Southern counterparts; ii) the latter collect data “in the field”, since Northern researchers are often not keen on accepting working conditions offered in the South (e.g. delays in payment, security issues, etc.); and iii) the data are subsequently analyzed by the former [18–20]. Researchers from the South may be invited to contribute substantially to the research or grant proposal application, but that is often because the funding agency requires their official participation. Typically, however, their involvement in later stages (decision-making or paper writing) remains limited. It is equally convenient to reach out to local researchers to shepherd proposals through countries’ research ethics board approval processes [21]. A fundamental part of the research takes place in Western institutions, while researchers from the poorer settings are given the major responsibility of managing the field work. In addition, ethical standards and managerial rules are set by Northern funders [21] and/or North-based scientific journals. For example, the latter do not acknowledge contributions that are considered trivial “technical tasks”, which are deemed of lesser value than designing the study [22]. Guidance on authorship order from journal editors “does not address or mitigate unfair practices which can occur in global health research due to power differences between researchers from high and low-middle income countries” [22].

This pattern of not overtly acknowledging inequality is also present at the individual level. Indeed, daily and visible inequalities (in terms not only of position in the research team and decision-making power, but also of living standards, access to information and resources, security and evacuation schemes, children’s schooling, etc.) between Southern researchers and Northern expatriates, and among Southern researchers themselves, all working together in resource-limited countries, are often not talked about. However obvious these inequalities may be, they are “not generally spoken about in professional engagements, although they might shape personal relationships and attitudes in everyday collaboration” [1]. In some cases, researchers from the South have voiced explicit complaints to challenge the status quo, but they have remained a minority [23]. The culture of “pretense”, that is, “unknowing what is known”, is widespread in partnerships between Northern researchers and sub-Saharan researchers, given that the latter are often unable to lead or to contribute substantially to scientific publications for lack of English-language skills, for instance [18]. Conversely, academics from sub-Saharan African countries report frustrations at not being consulted from the beginning of a research project, when the main conceptual issues are discussed, or at having to deal with young Northern researchers not adequately prepared for on-the-ground realities [24].

Geissler attempted to explain the lack of reference to these inequalities among research staff in public speeches and scientific writings: such transparent unpacking would “infringe the postulate of ‘equality-in-difference’ (as opposed to the recognition of unequal conditions and same human needs): Persons are (legally) equal but belong to different places and economic… orders” [1]. This narrative appears contradictory when considering the genuine charitable motivation that many Northern researchers share.

#### The promised land of scientific value

At the macro level, in higher-income countries, the past 30 years have seen a proliferation in university programs dedicated to global health. In this landscape, Western research universities hasten to create collaborations that can give their students and researchers opportunities to work in poor resource settings [21], and host institutions in the Global South are described as “partners”.

Partnerships between Northern and Southern institutions actually provide Western researchers with access to “desirable” patient populations [2]. Social science researchers have shown how Africa can actually become an opportune “uncontaminated” terrain for research, and for clinical research in particular [1, 2]. Indeed, there is a general perception that people in Western countries cannot be “virgin” research subjects because of their widespread use of pharmaceuticals [2]. Crane explicitly highlights an “easy access to the bodies of under-treated patients in… the global South” [2]. At the same time, at the meso level, it appears Northern research teams doing clinical work in the South do come with their own frames, yet they also have moral motives attached to them. For instance, American teams doing clinical research in West African countries on antiretroviral therapy also have displayed a genuine “moral and political project aimed at using science to ‘prove’ that Africans could indeed take the drugs properly, and should be given the opportunity to do so” [2].

#### A donor/recipient dynamic hard to overcome

As outlined above, there seems to be an inherent contradiction between how global health equality is currently framed in global discourses (which do not acknowledge the unequal conditions in which research is done) and the logic of charity still promoted by many Northern researchers. However, borrowing from humanitarian aid types of frames (i.e., caring for the poor) does not necessarily help (re)balance partnerships. Humanitarian aid is “motivated by the desire to relieve suffering and based on the ethics of a shared humanity” [25]. Yet history has shown there may be political purposes behind humanitarian work and charitable efforts [26]. In addition, charity actually tends to perpetuate aid dependency. The debate about aid dependency is, however, highly polarized; to understand what is at stake requires taking a broader perspective. Whether aid dependency is a reality or not, or whether we think there is too much aid or not enough, is actually not relevant to the debate on equal partnerships. What matters most is what meaning donors attach to foreign aid and how it is conceived. For instance, some scholars, such as Thomas Pogge, argue that moral reasons alone should compel Northern countries to repair the damages caused during colonization through foreign assistance [27]. However, conferring upon the aid system a simple, historical *raison d’être* can hardly provide the foundation for genuinely equal partnerships. Morality and charity are similar values: both take a paternalistic approach to foreign assistance by refusing to consider that countries and people are non-static entities [28]. Both therefore perpetuate donor–recipient dynamics that are incompatible with principles of partnerships.

Pragmatic concerns also perpetuate the imbalance. In global health partnerships, large sums of money are at stake. If an African university is willing to administer a U.S. grant that is “larger than its entire university budget” [2], the conditions for ensuring success and sustainability should be set by all parties ahead of time. Besides, if truly equitable partnerships are to take shape, then in determining overhead reimbursements each partner must be held equally accountable [2].

While partnerships often provide underfunded Southern research institutions with numerous types of assistance (renovation, equipment, infrastructure, employment, funds, etc.), these are often entwined with Western funders’ frames and expectations about program or intervention priorities. Often, at the macro level, Western understanding and conceptualization of global health issues also dominate. For instance, the globalization of the Western perspective on mental health knowledge has been questioned by scholars like Derek Summerfield [29]. The Lancet series on global mental health published in 2007, which drew together leading experts from the North, argued that every year nearly one-third of the world’s population will experience mental conditions [30]. The authors of this series urged the need for scaling up mental health services worldwide. According to Summerfield, these recommendations based on Western views, definitions, and solutions in mental health cannot be routinely applied to people in the South [29]. Mental illness still represents a taboo subject, sparking stigma in much of Africa. For example, a study in Uganda revealed that the Western definition of the term “depression” is not culturally acceptable among the population [31], while another in Nigeria found that people responded with fear, avoidance, and anger to those who were medically declared to have a mental illness [32]. Summerfield argues that, since the European Enlightenment, Western psychiatric science has sought to convert human pain, misery, and madness into technical and standardized terminologies that are universally valid and subject to interventions by Northern experts [33]. This point echoes Fassin’s work on dominant constructions [34] and corresponds to the classical argument of Edward Said, who postulated that local knowledge systems are routinely undermined and overruled by Northern experts and global knowledge [35].

It is worth pointing out that inequality does not only affect scientific partnerships. In global health practice, humanitarian aid and the field of global health typically mingle, the former thereby providing additional illustrations of unequal partnerships at the micro and meso levels. Research on humanitarian NGOs has described recurring situations in which international volunteers from the North come to countries in crisis for short periods of time, bringing with them structures and hierarchies that perpetuate unequal relationships with those remaining in the field [36]. There is also a broad lack of consideration for local experience. For instance, at the micro level, individuals working in refugee camps in Africa or the Middle East are not sufficiently invited by European-based practitioners to share their experience and lessons learned on how to manage refugee crises [37]. Sadly, knowledge translation paths seem to remain unchanged, i.e., streaming from North to South, conveying the impression that most Northern practitioners do not consider their Southern counterparts to be valuable sources of expertise, even though they have much more experience on the matter of coordinating the flow of refugees [38, 39]. Overall, moving up to a macro-level perspective, unless and until general aid governance evolves from paternalism to greater recognition of Southern expertise, it is doubtful that the donor–recipient dynamic will be able to change within research partnerships.

### A few proposals towards genuinely equal partnerships

#### Rethinking global health partnerships

Going beyond this rather negative picture, several concrete steps can be taken to rethink global health partnerships. At the macro level, this would involve rethinking what global health is—as academic field—mainly by being explicit about past and present inequalities between Northern and Southern universities that this field has so far ignored. To start with, Southern universities must be able to contribute substantially to redefining global health. This means ensuring greater representation of Southern research institutions at global health meetings such as CUGH along with a strong voice in global health governance decision-making forums (which would include implementing new rules for board elections and staff recruitment).

Second, at the micro and meso levels, it requires rethinking the meaning of partnership. Several authors (including from the South) have reflected critically on this notion and proposed frameworks to create conditions of equal research partnerships in global health [14, 40, 41]. Zarowsky suggests that “collaborative approaches… require (re)negotiation of everything from objectives to governance, and sensitivity to and respect for the… diverging agendas and constraints of various ‘stakeholders’” [41]. We concur with this author that ingredients of a successful partnership also entail “tolerance for disagreement, taking time to build and maintain trust…, attention… and the details of who participates in both financial and scientific decision making” [41].

At the individual level, academics from the North, heads of North-based research centres, and managers of funding institutions need to understand the history of global health. This would primarily involve knowing and valuing local specificities as well as past and present public health experiences and practices, recognizing material differences in equipment and living standards, acknowledging the legacy of paternalistic tropical medicine and international health practices, and understanding how Southern people’s memories of colonialist medicine still affect how they perceive global health projects. Indeed, as Larkan et al. argue, “social, cultural, environmental, and technological realities… shape and define successful research partnerships in global health” [14].

Yet only with sufficient cross-disciplinary and cultural training can one begin to develop an understanding of the local problems and research needs before actually conceiving the research project [42]. Such training would stimulate global health academics’ inclination to develop reflexivity and self-criticism. Besides understanding cultural complexities, we recommend developing and strengthening teamwork skills in cross-disciplinary and cross-category contexts (involving research users, such as policy-makers, as well as representatives of funding institutions and heads of research centres) and ensuring that scientifically produced results are useful and usable [4].

#### Young researchers from North and South: a promising avenue?

At the meso level, many global health research partnerships champion an unambiguous obligation to engage in capacity-building and offer research skills training to Southern researchers with the goal of fostering local expertise and leadership in global science. However, as we saw earlier, the inclusion of local researchers and host institutions as knowledge producers often remains superficial and insufficient to counteract the classic unidirectional North-to-South trend of global health knowledge transfer in sub-Saharan Africa and instead support a truly reciprocal, bi-directional flow [2, 24]. Furthermore, after being trained in Northern academic institutions, individual African researchers typically decide to stay in their host country [43]. At the micro level, classic proposals to achieve this ambitious transformation towards greater partnership equality include training young researchers from both North and South in more meaningful ways, having them interact with each other, and providing incentives to African researchers to return to their home countries.

While participating in and/or graduating from Northern universities’ interdisciplinary programs may represent a career boost for young Southern researchers, these achievements do not necessarily translate into better equilibrium in North–South collaborations. Indeed, from meso and micro perspectives, re-integrating into traditional discipline curricula in sub-Saharan African universities may be difficult for Southern researchers trained in the North [44]. Moreover, because their salaries are often lower than expected and/or because older researchers already hold senior positions, these young academics are often tempted to work as consultants for international projects or undertake political careers in government ministries [45]. These experiences do not contribute to creating more balance in global health research partnerships.

In addition, patterns of domination persist, as there are continued questions around who benefits from the research and who has access to the data [21]. There are still too many meso-level examples of “unilateral capacity building with limited benefits for the local community” [21]. Initiatives undertaken by the Working Group on Ethics in Global Health Training (WEIGHT) go in the right direction; they developed guidelines for students’ conduct of global health research in the field [46]. Still, as rightly pointed out by Hunt and Godard, the most difficult task is for global health students to “develop research projects that have the potential to generate locally applicable results and yield other benefits for the participants” [21]. To produce micro-level changes, the authors suggest creating additional training opportunities for local students and researchers “so that there is a greater chance of capacity building on all sides” [21]. Finally, practical considerations for transmitting research findings to participants and community members should be incorporated into research proposals from the beginning.

The Emerging Voices program of the Institute of Tropical Medicine (ITM) in Antwerp is an interesting example of an initiative yielding promising micro- and meso-level results. Launched in 2010, the Emerging Voices for Global Health (EV4GH) is a multi-partner blended training program for young researchers on health research and scientific communication, which creates an intensive learning and networking environment. It is aimed primarily at empowering health researchers from the Global South by providing intensive skills training, facilitating their participation in the global health arena, and providing a space where their voices can be heard [47]. Since its initiation by the ITM, and the first Global Symposium on Health Systems Research (GSHSR) in 2010 in Montreux, the program has been continuously moved to embrace many others institutions, mainly from the South, including the Institute of Public Health in Bangalore (India), the Peking University Health Science Centre (China), the University of Cape Town (South Africa), and the University of the Western Cape (South Africa). Of the five EV4GH programs held to date, four have been linked to the bi-annual GSHSR. Through a combination of distance learning and intensive face-to-face coaching, participants learn skills for making oral presentations at scientific conferences, communicating through social media, and publishing in scientific journals [47]. So far, the initiative has given voice to more than 240 young researchers from all over the world, but most importantly from the global South. In a step forward, EV4GH has recently reconfigured itself with a globally representative elected governing board and a new secretariat based at the Institute of Public Health in Bangalore.

The Consortium for Health Policy and Systems Analysis in Africa (CHEPSAA), which ran from 2011 to 2015, is another example of a beneficial meso-level initiative. It brought together 11 African and European university-based groups involved in teaching and research. CHEPSAA reportedly enabled the “deliberate sharing of experience across geographic and academic/policy boundaries, which generated practical benefits for both the southern and northern partners” [48]. Benefits notably included the development of innovative cross-disciplinary educational programs. More recent networks, like the Collaborative for Health Systems Analysis and Innovation (CHESAI), aim to develop “shared understandings and coproduce knowledge through joint writing” [48]. Programs like McMaster University’s International Pediatric Emergency Medicine Elective, which brings together medical students from Canada and abroad to study together in Canada [49], may also start to turn the tide towards collaborative approaches to global health education.

The next hurdle may be to connect all these training and networking initiatives to create shared visions of equal partnerships between North and South and among Southern young researchers. This would indeed be useful to reduce fragmentation and to learn from existing or past initiatives such as CHEPSAA.

Last but not least, at the macro level it is of utmost importance to continue advocating for substantial investments in global health research and the use of research evidence in the South. Without increased public domestic funding in these countries, inequalities in global health research partnerships will persist. Sub-Saharan Africa-based researchers need to engage with policy-makers and raise awareness on this issue.

## Conclusion

Encouraging discourse around partnership between Northern and Southern institutions has emerged as a key strategy for confronting (at least rhetorically) the problem of inequality [9]. However, the imbalances within global health research partnerships will persist for as long as the culture of consciously *unknowing* what is known continues unchallenged, and especially given the difficulty of moving away from charitable donor–recipient relationships.

Within this context, more equitable and transformative partnerships are vital to overcome the Northern influence on many partnerships that often provides a breeding-ground for inequality. On a more positive note, initiatives that involve cross-cultural and cross-disciplinary training, as well as training in global health ethics, are becoming increasingly visible, especially those involving young researchers.

## Abbreviations

CHEPSAA: Consortium for Health Policy and Systems Analysis in Africa
CHESAI: Collaborative for Health Systems Analysis and Innovation
CUGH: Consortium of Universities for Global Health
EV4GH: Emerging Voices for Global Health
GHSHR: Global Symposium on Health Systems Research
ITM: Institute of Tropical Medicine
NGOs: Non-Governmental Organizations
WEIGHT: Working Group on Ethics in Global Health Training

## References

[1] Geissler PW (2013). Public secrets in public health: knowing not to know while making scientific knowledge. Am Ethnol.

[2] Crane JT (2010). Unequal ‘partners’. AIDS, academia, and the rise of global health. Behemoth.

[3] Sheikh K, Bennett SC, el Jardali F, Gotsadze G (2016). Privilege and inclusivity in shaping Global Health agendas. Health Policy Plan.

[4] Ridde V, Hunt M, Dagenais C, Agier I, Nikiema A, Chiocchio F (2016). Une politique concernant les données issues d’un programme de recherches interventionnelles en santé mondiale [a policy regarding data generated by a global health intervention research program]. Bioéthique online.

[5] Murphy J, Hatfield J, Afsana K, Neufeld V (2015). Making a commitment to ethics in global health research partnerships: a practical tool to support ethical practice. J Bioeth Inq.

[6] Ridde V, Zerbo R, Yaogo M, Samb O, Faye A (2012). Les deux solitudes : les systèmes de recherche et d’organisation des soins de santé au Burkina Faso à travers l’histoire du paiement des soins par les patients. Working paper, Health Research capacity strengthening – global learning.

[7] Macfarlane SB, Jacobs M, Kaaya EE (2008). In the name of global health: trends in academic institutions. J Public Health Policy.

[8] Merriam-Webster Dictionary [online]. Definition of “deconstruct”; 2016. https://www.merriam-webster.com/dictionary/deconstruct. Accessed 29 Aug 2017.

[9] Koplan JP, Bond TC, Merson MH, Reddy KS, Rodriguez MH, Sewankambo NK (2009). Towards a common definition of global health. Lancet.

[10] Consortium of Universities for Global Health. Meeting report: university consortium for Global Health inaugural meeting. Consortium of Universities for Global Health, 2008.

[11] Youde J (2010). Biopolitical surveillance and public health in international politics.

[12] Beaglehole R, Bonita R. What is global health? Glob Health Action. 2010;3 10.3402/gha.v3i0.5142. 10.3402/gha.v3i0.5142 PMC2852240 20386617

[13] Frenk J, Gómez-Dantés O, Moon S (2014). From sovereignty to solidarity: a renewed concept of global health for an era of complex interdependence. Lancet.

[14] Larkan F, Uduma O, Lawal SA, van Bavel B (2016). Developing a framework for successful research partnerships in global health. Glob Health.

[15] Beran D, Byass P, Gbakima A, Kahn K, Sankoh O, Tollman S, Witham M, Davies J (2017). Research capacity building-obligations for global health partners. Lancet Glob Health.

[16] Geissler PW, Pool R (2006). Editorial: popular concerns about medical research projects in sub-Saharan Africa—a critical voice in debates about overseas research ethics. Tropical Med Int Health.

[17] White L (2000). Speaking with vampires: rumor and history in colonial Africa.

[18] Ouattara F, Ridde V (2013). Expériences connues, vécues… mais rarement écrites: à propos des relations de partenariat Nord-Sud. Nouvelles Pratiques Sociales.

[19] Hasnida A, Borst RA, Johnson AM, Rahmani NR, van Elsland SL, Kok MO (2017). Making health systems research work: time to shift funding to locally-led research in the south. Lancet Glob Health.

[20] Issac A (2017). Regional inequities in knowledge production – reflections from HSR 2016. Antwerp: international health policies network.

[21] Hunt MR, Godard B (2013). Beyond procedural ethics: foregrounding questions of justice in global health research ethics training for students. Glob Public Health.

[22] Smith E, Hunt M, Master Z (2014). Authorship ethics in global health research partnerships between researchers from low or middle income countries and high income countries. BMC Med Ethics.

[23] Nordling L (2012). African researchers sue flagship programme for discrimination. Nature.

[24] Ridde V, Capelle F (2011). La recherche en santé mondiale et les défis des partenariats Nord-Sud. Can J Public Health.

[25] Hilhorst DJ (2007). Saving lives or saving societies? Realities of relief and reconstruction.

[26] Fassin D, Inhorn MC, Wentzell EA (2006). That obscure object of global health. Medical anthropology at the intersections: histories, activisms, and futures.

[27] Pogge TW (2008). World poverty and human rights.

[28] Sanghera B (2016). Charitable giving and lay morality: understanding sympathy, moral evaluations and social positions. Sociol Rev.

[29] Summerfield D (2008). How scientifically valid is the knowledge base of global mental health?. BMJ.

[30] Horton R (2007). Launching a new movement for mental health. Lancet.

[31] Okello ES, Ekblad S (2006). Lay concepts of depression among the Baganda of Uganda: a pilot study. Transcult Psychiatry.

[32] Kabir M, Iliyasu Z, Abubakar IS, Aliyu MH (2004). Perception and beliefs about mental illness among adults in Karfi village, northern Nigeria. BMC Int Health Hum Rights.

[33] Summerfield D, Rosen G (2004). Cross-cultural perspectives on the medicalization of human suffering. Posttraumatic stress disorder: issues and controversies. Chichester.

[34] Fassin D, Comacchio C, Golden J, Weisz G (2008). AIDS orphans, raped babies, and suffering children: the moral construction of childhood in post-apartheid South Africa. Healing the world’s children: interdisciplinary perspectives on child health in the twentieth century.

[35] Said EW (1993). Culture and imperialism.

[36] Redfield P (2012). The unbearable lightness of expats: double binds of humanitarian mobility. Cult Anthropol.

[37] Verwimp P, Maystadt JF (2015). The European refugee crisis: what we can learn from refugees in sub-Saharan Africa.

[38] Rohwerder B (2016). Humanitarian response in middle-income countries. GSDRC helpdesk research report 1362.

[39] Ponthieu A, Incerti A (2016). Continuity of care for migrant populations in southern Africa. Ref Surv Q.

[40] Godoy-Ruiz P, Cole DC, Lenters L, McKenzie K (2016). Developing collaborative approaches to international research: perspectives of new global health researchers. Glob Public Health.

[41] Zarowsky C (2011). Global health research, partnership, and equity: no more business-as-usual. BMC Int Health Hum Rights.

[42] Krull W (2005). Editorial: helping to create symmetric partnerships: a new approach to supporting research in sub-Saharan Africa. Tropical Med Int Health.

[43] Muula AS (2005). Is there any solution to the “brain drain” of health professionals and knowledge from Africa?. Croat Med J.

[44] Kadio K (2015). Politiques publiques de santé fondées Sur les données probantes en Afrique: Aller au-delà des frontières disciplinaires pour répondre aux besoins et aux valeurs des populations. Cahiers Scientifiques REALISME.

[45] Olivier de Sardan JP (2011). Promouvoir la recherche face à la consultance: autour de l'expérience du LASDEL (Niger-Bénin). Cahiers D'Études Africaines.

[46] Crump JA, Sugarman J (2010). Working group on ethics guidelines for Global Health training (WEIGHT). Ethics and best practice guidelines for training experiences in global health. Am J Trop Med Hyg.

[47] Hercot D, Keugoung B, Zerbo A, Appelmans A, Van Damme W (2012). L’expérience « talents émergents en santé mondiale » : Une forme de renforcement intensif des capacités des jeunes chercheurs du Sud [the emerging voices for Global Health initiative: an intensive capacity-building effort for young researchers from the south]. Med Sante Trop.

[48] Sheikh K, Schneider H, Agyepong IA, Lehmann U, Gilson L (2016). Boundary-spanning: reflections on the practices and principles of Global Health. BMJ Glob Health.

[49] Valani R, Sriharan A, Scolnik D (2011). Integrating CanMEDS competencies into global health electives: an innovative elective program. CJEM.

